# Supplementary material for: Genome-wide CRISPR Screens in T Helper Cells Reveal Pervasive Crosstalk between Activation and Differentiation
Source: Cell. 2019 Feb 7;176(4):882–896.e18. doi: 10.1016/j.cell.2018.11.044 (PMC6370901; doi:10.1016/j.cell.2018.11.044)
Supplement: Data S2. Processed Data from All the Steps of the Analysis, Related to Figure 1 [file mmc2.zip › supplemental data/motif analysis/Pparg_homer/homerResults/motif2.similar.html]

motif2

## Information for motif2

G
A
C
T
T
A
C
G
T
G
C
A
G
T
A
C
G
A
T
C
G
A
C
T
G
A
C
T
G
A
C
T
T
A
C
G
G
T
C
A
G
A
T
C
G
A
T
C
  
Reverse Opposite:  

C
T
A
G
C
T
A
G
C
A
G
T
A
T
G
C
C
T
G
A
C
T
G
A
C
T
G
A
C
T
A
G
C
A
T
G
A
C
G
T
A
T
G
C
C
T
G
A
  

|  |  |
| --- | --- |
| p-value: | 1e-1031 |
| log p-value: | -2.375e+03 |
| Information Content per bp: | 1.610 |
| Number of Target Sequences with motif | 3170.0 |
| Percentage of Target Sequences with motif | 25.32% |
| Number of Background Sequences with motif | 2186.7 |
| Percentage of Background Sequences with motif | 6.10% |
| Average Position of motif in Targets | 99.5 +/- 48.1bp |
| Average Position of motif in Background | 101.2 +/- 62.0bp |
| Strand Bias (log2 ratio + to - strand density) | 0.1 |
| Multiplicity (# of sites on avg that occur together) | 1.09 |
| Motif File: | file (matrix) reverse opposite |

### Similar de novo motifs found

|  |  |  |  |  |  |  |  |
| --- | --- | --- | --- | --- | --- | --- | --- |
| Rank | Match Score | Redundant Motif | P-value | log P-value | % of Targets | % of Background | Motif file |
| 1 | 0.916 | T G C A T G A C A G T C A G C T G A C T A G C T T A C G T G A C T G A C G T A C | 1e-703 | -1620.174627 | 14.51% | 2.80% | motif file (matrix) |
| 2 | 0.713 | A C G T C T A G A C T G A C T G A C G T A T G C C G T A C T G A C T G A C T A G C A T G A C G T | 1e-571 | -1315.125408 | 11.89% | 2.29% | motif file (matrix) |
| 3 | 0.898 | C A T G A G C T T A C G G T C A G T A C A G T C A G C T G C A T A C G T T C A G | 1e-514 | -1185.205536 | 19.63% | 6.55% | motif file (matrix) |
| 4 | 0.731 | G A T C G A C T G A C T G A C T T A C G T G C A G A T C G T A C G A C T T G C A | 1e-484 | -1115.606751 | 15.85% | 4.67% | motif file (matrix) |
| 5 | 0.885 | A G C T T C A G G T C A G T A C A G T C A C G T C G A T A C G T | 1e-460 | -1061.496452 | 17.17% | 5.59% | motif file (matrix) |
| 6 | 0.765 | T G C A G T A C A G T C A G C T A G C T G C A T C T A G A T C G A G T C G T A C A G C T C T G A | 1e-295 | -680.689826 | 7.05% | 1.53% | motif file (matrix) |
| 7 | 0.744 | G A T C G A C T A G C T G A C T T A C G T G A C G A T C T G A C | 1e-244 | -562.633289 | 28.94% | 16.88% | motif file (matrix) |
| 8 | 0.748 | A C T G A T G C A G T C C G T A T G C A C G T A A C T G A C T G | 1e-100 | -231.572384 | 8.12% | 3.91% | motif file (matrix) |
| 9 | 0.619 | T C G A C T G A C T A G A T C G A C T G A C G T C T A G T G C A A T G C C G T A | 1e-64 | -148.020596 | 2.67% | 0.90% | motif file (matrix) |
